# Coexistence of degenerative aortic stenosis and wild type transthyretin-related cardiac amyloidosis: a potentially dangerous association that can be non-invasively identified

**DOI:** 10.1186/1750-1172-10-S1-P45

**Published:** 2015-11-02

**Authors:** Simone Longhi, Agnese Milandri, Christian Gagliardi, Massimiliano Lorenzini, Francesco Saia, Ornella Leone, Pier Luigi Guidalotti, Claudio Rapezzi

**Affiliations:** 1 Department of Experimental, Diagnostic and Specialty Medicine – DIMES, Alma Mater Studiorum, University of Bologna, Cardiology, 40138, Bologna, Italy; 2 Department of Pathology, Sant'Orsola-Malpighi Hospital, Pathology, 40138, Bologna, Italy; 3 Department of Experimental, Diagnostic and Specialty Medicine – DIMES, Alma Mater Studiorum, University of Bologna, Nuclear Medicine Unit, 40138, Bologna, Italy

## Background

Degenerative aortic stenosis (AS) and wild type transthyretin (TTR)-related cardiac amyloidosis (wt-ATTR) share a common demographic and clinical profile. It has been recently suggested that the coexistence of wt-ATTR could negatively influence the outcome of elderly patients with aortic stenosis undergoing transcatheter aortic valve replacement (TAVR). TTR-related cardiac amyloidosis can be accurately identified by technetium-99m-3, 3-diphosphono-1, 2 propanodicarboxylic acid (99mTc-DPD) scintigraphy. We decided to investigate the coexistence of cardiac amyloidosis in elderly patients with aortic stenosis referred for aortic valve replacement (TAVR or surgery).

## Methods

Since October 2014 we prospectively evaluated with 99mTc-DPD scintigraphy all patients diagnosed with degenerative AS and one or more of the following: paradoxical low flow-low gradient severe AS, QRS voltage-left ventricular (LV) wall thickness mismatch, echocardiographic findings suggestive of myocardial infiltrative disease (increased thickness of atrio-ventricular valves or interatrial septum or right ventricular free wall, pericardial effusion, granular sparkling of ventricular myocardium). Cases with intense myocardial tracer uptake underwent endomyocardial biopsy (EMB).

## Results

Five out of 42 patients underwent 99mTc-DPD scintigraphy that showed strong myocardial uptake in all. EMB demonstrated TTR-related amyloid infiltration in all cases. Genetic analysis excluded TTR gene mutations; so wt-ATTR was diagnosed. Median age was 88 (range 86-91), 3/5 were males. Two had a history of carpal tunnel syndrome and all were symptomatic for exertional dyspnoea (NYHA class III-IV). At echocardiography mean LV wall thickness was 18±2 mm, LV ejection fraction was 54±10% (38%-64%). Functional aortic valve area was between 0.4 and 0.9 cm2;one case had a low flow-low gradient and reduced LV ejection fraction (38%); maximum aortic gradient in the other 4 cases was 59±30 mmHg. Atrio-ventricular valve thickening was present in all, and mild pericardial effusion was present in 3 cases. Tissue Doppler S wave was reduced in all cases. QRS voltage was normal in one and increased in 4 patients.

## Conclusion

Coexistence of degenerative AS and wt-ATTR cardiac amyloidosis (a potentially dangerous condition in patients undergoing AVR or TAVR) can be suspected by clinical and echocardiographic elements and effectively diagnosed by 99mTc-DPD scintigraphy.

